# Evolution of impulsive–compulsive behaviors and cognition in Parkinson’s disease

**DOI:** 10.1007/s00415-019-09584-7

**Published:** 2019-10-18

**Authors:** Aleksander H. Erga, Guido Alves, Ole Bjørn Tysnes, Kenn Freddy Pedersen

**Affiliations:** 1 The Norwegian Centre for Movement Disorders, Stavanger University Hospital, P.O. Box 8100, 4068 Stavanger, Norway; 2 Department of Neurology, Stavanger University Hospital, Stavanger, Norway; 3 Department of Chemistry, Bioscience and Environmental Engineering, University of Stavanger, Stavanger, Norway; 4 Department of Clinical Medicine, University of Bergen, Bergen, Norway; 5 Department of Neurology, Haukeland University Hospital, Bergen, Norway

**Keywords:** Parkinson’s disease, Impulse control disorders, Cognition, ParkWest

## Abstract

The longitudinal course of ICBs in patients with Parkinson’s disease (PwP) relative to controls has not been explored as of yet. The aim of this study is to determine the frequency, evolution and associated cognitive and clinical features of impulsive and compulsive behaviors (ICBs) over 4 years of prospective follow-up in a population-based cohort with early Parkinson’s disease (PD). We recruited 124 cognitively intact participants with early PD and 156 matched controls from the Norwegian ParkWest study. ICBs were assessed using the self-report short form version of the Questionnaire for Impulsive–Compulsive Disorders in PD. Cognitive changes were examined in PwP with and without ICBs who completed the 4-year follow-up. Generalized linear mixed modelling and mixed linear regression were used to analyze clinical factors and cognitive changes associated with ICBs in PwP over time. ICBs were more common in PwP than controls at all visits, with an age-adjusted odds ratio (OR) varying between 2.5 (95% CI 1.1–5.6; *p* = 0.022) and 5.1 (95% CI 2.4–11.0; *p* < 0.001). The 4-year cumulative frequency of ICBs in PwP was 46.8% and 23.3% developed incident ICBs during the study period, but the presence of ICBs was non-persistent in nearly 30%. ICBs were independently associated with younger age (OR 0.95, 95% CI 0.91–0.99: *p* = 0.008) and use of dopamine agonist (OR 4.1, 95% CI 1.56–10.69). Cognitive changes over time did not differ between patients with and without ICBs. In conclusion, ICBs are common in PwP, but are often non-persistent and not associated with greater cognitive impairment over time.

**Electronic supplementary material:**

The online version of this article (10.1007/s00415-019-09584-7) contains supplementary material, which is available to authorized users.

## Introduction

Impulsive and compulsive behaviors (ICBs) are common complications of dopaminergic replacement therapy (DRT) in Parkinson’s disease (PD), affecting between 13 and 30% of patients [10, 34]. ICBs encompass several addiction-like disorders related to reward-based activities, such as gambling, sexuality, shopping and eating. In addition, patients also report behavioral subtypes that are uncommon in the general population, such as hobbyism, punding, walkabout and dopaminergic medication overuse.

ICBs are more prevalent in people with PD (PwP) than normal controls (NCs) [10], and the initiation of DRT—particularly dopamine agonists (DAs)—has been suggested as the main risk factor for development of these symptoms [19]. This is supported by several observations, for example, similar frequencies of ICBs reported in clinical studies of drug-naïve PwP and NCs, presence of ICBs in medicated PwP is associated with DA use in several cohorts, and increased risk-taking behavior among DA users in experimental “on” state studies [2, 3, 6, 10, 19, 30, 34]. ICBs are often seen in young PwP, probably because DA is more often prescribed in younger versus older PwP during the initial phases of PD treatment [17, 31, 34]. DAs are often down-titrated or discontinued during the course of PD due to the emergence of side effects, like nausea, sleep disorders or dyskinesia. Consequently, the frequency of ICBs could be expected to decrease over time. However, this hypothesis has only been explored empirically in one previous publication, highlighting the need for further studies, and in particular studies that include controls [7].

ICBs have been associated with altered performance on executive tasks like set-shifting, reward-related decision making and concept formation [17, 26]. These findings are further supported by studies using neuroimaging, that demonstrate neuroanatomical differences and disrupted functional brain connectivity in mesolimbic and frontostriatal areas crucial for affective and reward processing [18]. Other cognitive domains, such as memory, visuospatial functioning, attention and language seem to be unaffected by ICBs, although this has been contested [26]. These observations have received support from a recent longitudinal study of cognition in PwP with ICBs [27], demonstrating that PwP and ICBs have relatively preserved executive functioning compared with PwP without ICBs over a mean follow-up period of 3.5 years. Similar findings were evident across several other cognitive domains, especially working memory [26].

The evolution of ICBs in PD is largely unknown, especially in later PD stages [2, 4, 7, 15, 16, 27, 28]. So far, no longitudinal studies have included controls and only one [27] had cognitive measures at follow-up. In addition, most studies were convenience samples, thereby limiting the generalizability of their findings. Thus, further studies using well-designed, population-based cohorts are needed. In the present study, we aim to (1) determine the longitudinal course and incidence of ICBs in PwP and controls, (2) examine associated clinical factors of ICBs in PD over time, and (3) describe the cognitive changes of PD patients with ICBs during follow-up, in a clinically well-characterized and population-based cohort.

## Methods

### Study design

PwP and controls were recruited from the Norwegian ParkWest study, an ongoing population-based, prospective cohort study of the incidence, neurobiology and prognosis of PD. A full overview of the diagnostic and recruitment procedures has been published elsewhere [1]. In short, a comprehensive strategy for case ascertainment was used to recruit a population-based sample of 212 participants with incident PD and 205 controls from four counties in Western Norway were enrolled in the study between November 1st, 2004, and August 31st, 2006. After baseline assessment, movement disorders neurologists initiated dopaminergic medication and evaluated participants clinically every 6 months. In addition, both PwP and controls were followed prospectively using standardized examinations of neuropsychiatric and cognitive functioning. This evaluation schedule was completed at baseline, 1 year follow-up, and thereafter every other year. Of 196 drug-naïve PwP at baseline, 20 were re-diagnosed during follow-up. Also, three controls developed incident PD during the follow-up period, and were excluded. Evaluation of ICBs was first introduced 5 years after the baseline visit, wherein 129 PwP without dementia and 160 controls without dementia participated. Of these, five PwP and four controls did not respond to ICB measures, yielding a cohort of 124 PwP and 156 controls eligible for this longitudinal study of ICBs. All PwP met the United Kingdom PD Society Brain Bank criteria for PD [14]. Signed written informed consent was obtained from all participants. The study was approved by the Regional Committee for Medical and Health Research Ethics, Western Norway.

### Procedures

All participants underwent a standardized examination program administered by trained members of the ParkWest study group. Information on demographic variables, lifestyle factors, clinical history and medication were gathered using semistructured interviews. Motor severity and disease stage were assessed by the Unified PD Rating Scale (UPDRS) and Hoehn and Yahr scale, respectively. Levodopa equivalent doses (LEDs) were calculated according to published recommendations [29].

Presence of ICBs was assessed using the self-report short form version of the Questionnaire for Impulsive–Compulsive Disorders in PD (QUIP) [33]. In accordance with published recommended cutoff scores, participants with positive responses to one or more screening questions of the QUIP were classified to have ICB.

Depressive symptoms was assessed using the Montgomery and Aasberg Depression Rating Scale (MADRS) [20]. Global cognitive function was assessed using the Mini-Mental State Examination (MMSE) [11]. In addition, a neuropsychological test battery was administered to assess cognitive functioning in four domains: (1) executive functioning (Semantic verbal fluency test [5] and Stroop interference condition [12]) (2) verbal memory (immediate recall, short-delay recall and long-delay recall from the California Verbal Learning Test II [8]) (3) visuospatial skills (Silhouette and Cube subtests of the Visual Object and Space Perception Battery [32]), and (4) attention (Stroop word reading and color naming test [12]). Both PwP and controls underwent cognitive testing at follow-up. A composite score for each domain was calculated as the average of the test scores after conversion into percent of maximum possible (POMP) scores, of which the maximum values were defined according to the maximum test scores of the NC group and the minimum values were set to zero. PD-associated dementia was diagnosed according to published criteria [9].

### Categorizing of ICBs

Presence and development of ICB symptoms for PwP were categorized as follows: (1) no ICBs include patients who never reported ICBs during the study period (2) persistent ICBs include those who reported ICBs at two or more following visits, including the last follow-up (3) fluctuating ICBs include patients who reported at least one reversion from ICBs to no ICBs, and (4) uncategorized ICBs include those with incident ICBs at the last visit only (either 2 or 4 years of follow-up).

### Statistical analyses

All statistical analyses were conducted using IBM SPSS Statistics for Windows, Version 24.0.0.1 (Armonk, NY: IBM Corp.). Group differences were analyzed using *t* tests and Mann–Whitney tests for continuous variables, and Pearson *χ*^2^ tests for categorical variables. Two-tailed *p* values < 0.05 were considered statistically significant.

Age-adjusted odds ratios (ORs) with 95% confidence intervals (CIs) for ICBs at each time of measurement were calculated using logistic regression. In these analyses, ICB status was used as dependent variable, while participant (PD or NC) and age was entered as independent variables.

Generalized linear mixed modelling (GLMM) was performed to investigate factors associated with ICBs in PD over time. The main parameter of interest was the fixed effect of DA use over time. In this analysis, ICB status was used as dependent variable and age, sex, disease duration, DA use and follow-up time were fitted as fixed effects. For repeated measures (ICB status), a scaled identity covariance structure was assumed, as this covariance structure yielded the least amount of error and best model fit [evaluated using Akaike’s information criteria (AIC) and Schwarz’s Bayesian criterion (BIC)]. Random intercept and slope were also included as they enhanced the model fit. A similar model using total LED instead of DA use was also fitted.

Neuropsychological performance was analyzed for patients that remained in the study after 4 years (*n* = 92), using mixed linear regression with scaled identify correlational structure. In these analyses, neuropsychological performance was used as dependent variable and age, sex, follow-up time and ICB status were used as independent variables.

## Results

### Participant characteristics

Characteristics of patients and controls at initial ICB assessment have been presented previously [10], and are summarized in Table 1. Briefly, patients had less education and demonstrated lower MMSE and higher MADRS scores than the NC group.

**Table 1:** Demographic and clinical characteristics at initial ICB assessment

Characteristics	PwP (*N* = 124)	Controls (*N* = 156)	*p* value
Age	70.4 (9.3)	70.2 (9.1)	0.864
Male, *n* (%)	76 (61.3)	82 (52.6)	0.144
Education, years	11.3 (3.3)	12.1 (3.5)	**0.042**
UPDRS motor score	22.7 (10.7)	–	–
PD duration, years	7.4 (1.7)	–	–
MMSE score	27.8 (2.5)	28.8 (1.5)	**0.001**
MADRS score	3.8 (4.4)	1.5 (2.9)	**0.001**
DA users, *n* (%)	77 (62.1)	–	–
Levodopa users, *n* (%)	102 (82.2)	–	–
Total LED^a^	612.7 (346.2)	–	–
DA LED^a^	182.5 (179.7)	–	–

Bold indicates *p* values < 0.05

*PwP* people with Parkinson’s disease, *UPDRS* Unified Parkinson’s Disease Rating Scale, *MMSE* Mini-Mental State Examination, *MADRS* Montgomery and Aasberg Depression Rating Scale, *DA* dopamine agonist, *LED* levodopa equivalent dosage

^a^Among DA users; patients using only levodopa were excluded from analysis

^b^Among levodopa users; patients using only DA were excluded from analysis

### Study flow

The flow of participants is available in online resource 1. Of 124 PwP and 156 controls, 17 PwP (3 withdrew and 14 died) and 20 controls (9 withdrew and 11 died) were lost to follow-up. A total of 22 PwP were diagnosed with PD dementia during follow, and 1 patient and 2 controls were excluded due to missing data.

### Evolution and course of ICBs

**Fig. 1:**
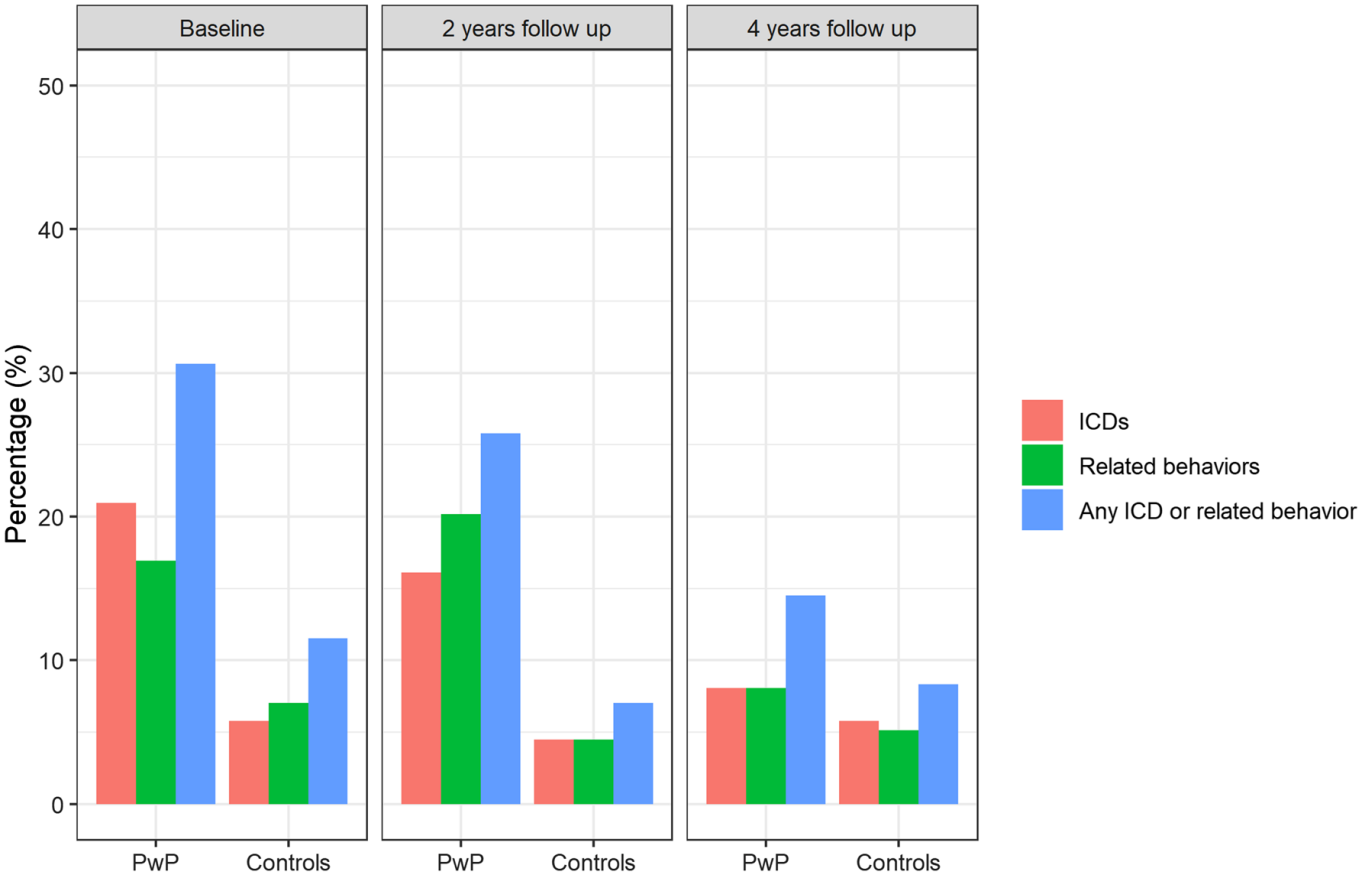
Frequencies of ICDs and related behaviors among patients with PwP and controls during the course of four years. *ICD* impulse control disorders, *PwP* people with Parkinson’s disease

Frequency of ICBs for patients and controls during the study period is presented in Fig. 1. Compared to controls, patients displayed more ICBs at each visit. The age-adjusted OR ranged from 3.4 (95% CI 1.8–6.5; *p* < 0.001) at initial assessment, to 5.1 (95% CI 2.4–11.0; *p* < 0.001) after 2 years, and 2.5 (95% CI 1.1–5.6; *p* = 0.022) at the 4-year follow-up. During the 4-year follow-up, 58 of 124 patients (46.8%) and 28 of 156 controls (17.9%) reported ICBs, yielding an age-adjusted OR of 4.2 (95% CI 2.4–7.4; *p* < 0.001). Overall, 20 of 86 patients (23.3%) and 10 of 138 controls (7.2%) developed incident ICBs during the 4-year study period, corresponding to an OR of 4.3 (95% CI 1.9–9.8; *p* < 0.001). Multiple ICBs were reported by 8.9% (11/124) of patients and 1.3% (2/156) of controls at initial assessment, 13.3% (14/105) and 2.2% (3/136) at 2-year follow-up, and 3.7% (3/82) and 3.2% (4/126) at 4-year follow-up, respectively. The frequencies of the individual ICB types are available in the online resource 2.

**Fig. 2:**
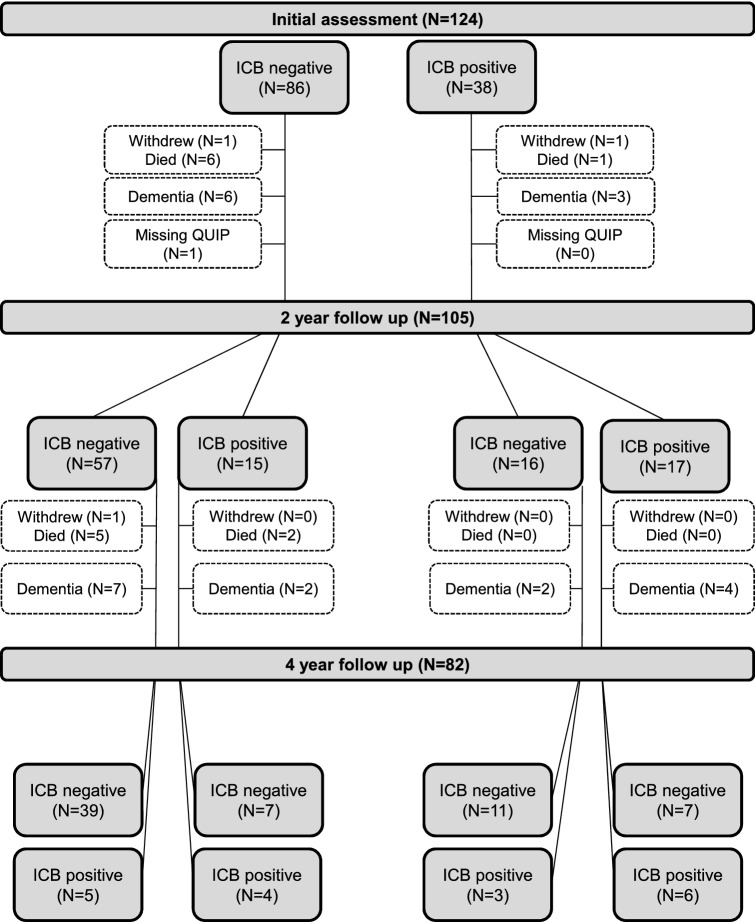
Flow chart of patients, stratified according the ICB status. *ICB* impulsive–compulsive behaviors

The course for different ICB categories in patients is presented in Fig. 2. A total of 105 patients had at least one follow-up visit after the initial assessment. Of these, 49.5% never reported ICBs, 13.3% had persistent and 28.6% non-persistent symptoms, while 8.6% reported ICBs at the last visit only. A detailed presentation of the occurrence and development of ICBs is presented in online resource 3.

### Associated clinical features

The cross-sectional association between ICB status and demographic and clinical variables is summarized in Table 2. In brief, ICB status was associated with lower age at each visit. DA use was associated with ICB status at the initial assessment and 2-year follow-up, but not at 4 years of follow-up. MADRS score was associated with ICB status at the initial assessment only.

**Table 2:** Clinical features stratified by ICB status in PwP during follow-up

Characteristics	Baseline (*N* = 124)		2-year follow-up (*N* = 105)		4-year follow-up (*N* = 82)	
	ICBs (*N* = 38)	Non-ICBs (*N* = 86)	ICBs (*N* = 32)	Non-ICBs (*N* = 73)	ICBs (*N* = 18)	Non-ICBs (*N* = 64)
Age, year	67.9 (7.7)	71.5 (9.8)*	66.7 (8.7)	72.9 (8.6) **	68.4 (10.1)	73.8 (8.7) *
Male, *n* (%)	26 (68.4)	50 (56.3)	21 (67.7)	38 (51.4)	13 (72.2)	31 (48.4)
UPDRS motor score	23.8 (10.5)	22.3 (10.8)	21.9 (9.7)	23.2 (11.6)	25.6 (13.2)	24.6 (12.7)
Dyskinesia^a^, *n* (%)	8 (21.1)	10 (11.6)	10 (31.3)	16 (22.0)	5 (27.8)	24 (37.5)
PD duration, year	7.4 (1.6)	7.3 (1.8)	9.3 (1.6)	9.3 (1.7)	11.4 (2.2)	11.1 (1.5)
MADRS score	5.4 (5.1)	3.1 (3.9) *	5.6 (5.7)	4.4 (4.5)	4.8 (7.2)	5.3 (5.0)
DA users, *n* (%)	32 (84.2)	45 (52.9) **	25 (68.8)	37 (50.7) *	10 (55.6)	33 (51.6)
Levodopa users, *n* (%)	29 (76.3)	74 (85.1)	28 (87.5)	67 (91.8)	18 (100.0)	57 (89.1)
Total LED^b^	731.2 (342.0)	658.2 (282.3)	956.9 (507.6)	798.5 (324.0)	1195.1 (527.9)	936.2 (410.9)
DA LED^b^	293.7 (132.4)	285.9 (149.7)	316.1 (131.1)	326.3 (142.7)	267.2 (154.3)	333.8 (132.0)
Levodopa dose^c^	505.2 (279.1)	410.3 (268.3)	590.2 (327.4)	485.2 (262.2)	634.7 (386.9)	544.7 (282.3)

*PwP* people with Parkinson’s disease, *ICB* impulsive and compulsive behaviors, *UPDRS* Unified Parkinson’s Disease Rating Scale, *MADRS* Montgomery and Aasberg Depression Rating Scale, *LED* levodopa equivalent dose, *DA* dopamine agonist. Univariate group differences indicated by: *p < 0.05; **p < 0.001

^a^Dyskinesia as defined by score ≥ 1 on UPDRS item 32

^b^Among DA users; patients using only levodopa were excluded from analysis

^c^Among levodopa users; patients using only DA were excluded from analysis

Longitudinal analysis using GLMM showed significant fixed effects of age (*F*(1,6) = 7.0, *p* = 0.008) and DA use (*F*(1,6) = 8.2, *p* = 0.004), see Table 3 for a summary of this model. DA use was associated with increased risk of ICBs with an estimated OR of 4.1 (95% CI 1.56–10.69; *p* = 0.004), while higher age was associated with lower risk of ICBs (OR 0.95, 95% CI 0.91–0.99; *p* = 0.008). ICBs were not associated with PD duration, sex or follow-up time. The fixed estimate for the interaction time × DA use was not significant. This model predicted 86.8% of ICB cases. Repeating this model with levodopa use instead of DA use did not demonstrate a significant effect of levodopa, while lower age remained significantly associated with ICB status.

**Table 3:** Effect estimates of generalized mixed regression models with ICB status as dependent variable

Outcome	Effects	OR	SE	95% CI for OR
ICB status	Main effects group			
	Sex^a^	− 0.31	0.39	(− 1.08, 0.45)
	Age	− 0.06**	0.02	(− 0.10, − 0.02)
	PD duration	0.10	0.11	(− 0.11, 0.31)
	Time	0.06	0.14	(− 0.22, 0.34)
	DA use^b^	1.41**	0.49	(0.44, 2.37)
	Interaction, time × DA use			
	Time × DA use^b^	− 0.29	0.17	(− 0.63, 0.05)
	Random effects			
	Residual effect	0.65***	0.07	(0.53, 0.79)
	Random intercept	1.95***	0.54	(1.13, 3.36)

*ICB* impulsive and compulsive behaviors, *DA* dopamine agonist, *PD* Parkinson’s disease, *SE* standard error, *CI* confidence interval

*p ≤ 0.05; **p ≤ 0.01; *p* ≤ 0.001

^a^With male gender set as reference category

^b^With “no DA use” set as reference category

### Cognitive features

Change in MMSE and POMP scores for all four cognitive domains was not associated with ICB status over time. A detailed account of the results of each mixed linear regression model is available in the supplemental material (E-4).

## Discussion

In this prospective longitudinal study of a population-based cohort of patients with PD, 47% of patients reported ICBs during the 4-year follow-up period. The 4-year cumulative incidence of ICBs was 23%. Occurrence of ICBs was consistently higher in patients with PD, with a more than fourfold increased risk of ICBs in PD compared to well-matched controls during follow-up. Among patients with at least one follow-up visit, ICBs resolved in nearly 30%, while 13 % had persistent symptoms during follow up. Presence of ICBs was associated with DA use and younger age, but not with greater cognitive decline over time. Although these findings demonstrate that ICB symptoms often resolve over time, they also underscore the need for continued clinical assessments of ICBs during the course of PD, as incident ICBs are also observed in the later stages of the disease.

The prevalence of ICBs decreased from around 30% at initial assessment to 22% after 4 years. During the same follow-up period, the proportion of DA users decreased about 10% and the proportion of levodopa users increased almost equivalently. These findings are consistent with a recent multicenter open-label trial reporting alleviation of ICBs in PD patients 12 weeks after switching from DAs to levodopa/carbidopa slow-release formulations. Still, more than half of our patients with ICBs were using DA at every visit. Possible explanations for this include underreporting of ICB symptoms in clinical practice and motor worsening or withdrawal syndrome during tapering or discontinuation of DA therapy [24]. Unfortunately, our study was not designed to address this issue. As opposed to the present study, one large longitudinal multicenter study recently showed that ICB prevalence increased from around 20% to nearly 33% after 5 years of follow-up [7]. However, this PD cohort was characterized by a high prevalence of DA treatment, which may also explain the high 5-year cumulative incidence of ICB around 46%. In comparison, the cumulative incidence of ICB in our cohort was about 50% lower after 4 years of follow-up.

To our knowledge, this is the first longitudinal study of ICBs in PD that includes NCs. We found a more than fourfold increased risk of prevalent and incident ICBs during the 4-year follow-up period. This finding support the results of a recent meta-analysis showing that PD patients have a twofold increased risk of ICBs compared with controls [19].

Our data confirm and expand numerous previous findings from cross-sectional [2, 34] and longitudinal studies reporting a strong association between DA usage and presence of ICBs in PD [4, 7, 15, 16, 27, 28]. Although our findings argue that the association between ICBs and DA use may be a class effect, clinical experience and long-term studies indicate a dose–effect relationship between ICBs and DAs [7], and the first treatment option is often to reduce DA dosage while stepping up the dosage of levodopa [16, 35]. Although some previous cross-sectional studies suggest an association between levodopa and ICBs [34], other longitudinal studies [4, 7], including ours, do not confirm this assumption. One should keep in mind that ICBs probably resolve slowly after DAs are discontinued, and that this may erroneously suggest an association between levodopa and ICBs even though ICBs appeared before levodopa was started or doses increased [7]. Also, for some patients discontinuation of DA might be necessary to ensure alleviation of ICB symptoms.

Even though the frequency of ICBs may diminish over time, there are still ample reasons for continued clinical screening of ICBs during the course of PD. As demonstrated recently, time to onset of ICB symptoms varies greatly following DA treatment [4]. In our study, new cases of ICBs emerged more than 5 years after PD was diagnosed. However, ICB presence was generally unstable and we cannot exclude that some patients with incident ICBs may have experienced such symptoms before the start of the present study. As such, our ICB remission rate is probably an underestimation. Other studies have reported substantially higher ICB remission rates, but this seems to vary considerably due to differences in sample sizes and methodological approaches [16]. Other explanations for the non-persistent nature of ICBs include the risk identifying sub-syndromal ICBs when utilizing QUIP, a methodological issue that could be addressed by also administering QUIP to the caregivers of the patients [23].

Our data suggest that ICB status in not related to greater cognitive impairment over time. Although these findings conflict with several studies investigating specific dopamine-sensitive executive functions, like risk assessment and decision making [6, 30], the lack of cognitive dysfunctions in other cognitive domains is consistent with reports from other cohorts investigating the association between ICBs and global cognitive functioning [3, 27, 31]. Data obtained after dementia onset were excluded in those who developed PD dementia during follow-up. This procedure could skew the estimates of POMP scores over time and thereby underestimate the cognitive decline of patients with ICBs. However, the rate of incident dementia at follow-up was not different between ICB-positive and ICB-negative patients at study start (data not shown), arguing that self-censoring of demented patients did not affect the results. This is further supported by the association between ICBs and younger age in our study.

Preserved cognitive functioning in patients with ICBs is also of clinical importance, especially for potential development of new management strategies for ICBs in PD. The current management strategy for ICBs is alterations of DRT, an approach not viable in all cases [36]. Alternative treatments have been suggested, including cognitive behavioral therapy [21, 22], a psychotherapeutic treatment commonly used in the treatment of ICBs in the general population [13, 25]. Although promising, the efficacy of CBT is contingent on relatively preserved cognitive functioning. Thus, the findings of this study provides an argument for continued research and development of CBT for PwP and ICBs.

The major strengths of our study include the population-based design, limited attrition during follow-up, well-characterized PD cohort and inclusion of controls from the same geographical area. There are also some limitations of this study. First, the sample is limited in size. Although this issue could result in less statistical power, the use of conservative statistical procedures decreases the risk of type II errors. Second, the use of QUIP may overestimate the frequency of ICBs. This issue has been highlighted in several previous publications, and the inclusion of semistructured interviews would probably result in lower risk of false positives. Still, the risk of inflated frequency estimates is similar in PwP and controls, and would therefore not influence the ORs of this study. In addition, the frequency estimates of this study are comparable to other studies gauging the full scope of ICBs. Finally, the neuropsychological test battery utilized in this study provides limited insights into specific executive functions previously associated with ICBs, such as risk assessment and set-shifting. However, this study provides valuable insights into several global indices of cognitive functioning, such as verbal memory, attention and visuospatial functioning. These cognitive domains are important determinants of global cognitive functioning, and essential when assessing cognitive decline in PwP.

## Electronic supplementary material

Below is the link to the electronic supplementary material.

Supplementary file1 (PDF 738 kb)
