# Supplementary material for: Evolution of impulsive–compulsive behaviors and cognition in Parkinson’s disease
Source: J Neurol. 2019 Oct 18;267(1):259–66. doi: 10.1007/s00415-019-09584-7 (PMC6954890; doi:10.1007/s00415-019-09584-7)
Supplement: Supplementary file 1 — Supplementary file1 (PDF 738 kb) [file 415_2019_9584_MOESM1_ESM.pdf]

Online resources for "Evolution of impulsive compulsive behaviors and cognition in Parkinson's disease", Journal of Neurology, Aleksander H. Erga[a*]; Guido Alves[a,b,c]; Ole Bjørn Tysnes[d,e]; Kenn Freddy Pedersen[a,b]

[a] The Norwegian Centre for Movement Disorders, Stavanger University Hospital, Stavanger, Norway

[b] Department of Neurology, Stavanger University Hospital, Stavanger, Norway

[c] Department of Chemistry, Bioscience and Environmental Engineering, University of Stavanger, Stavanger, Norway

[d] Department of Clinical Medicine, University of Bergen, Bergen Norway

[e] Department of Neurology, Haukeland University Hospital, Bergen, Norway

[*] Correspondance to aleksander.erga@sus.no

Online resource 1

Title Flow chart of patients with PD and controls in the Norwegian ParkWest study

Footnotes: PD=Parkinson's disease; PwP=People with Parkinson's disease; QUIP= Questinnaire of Impulsive and Compulsive behaviors in Parkinson's disease.

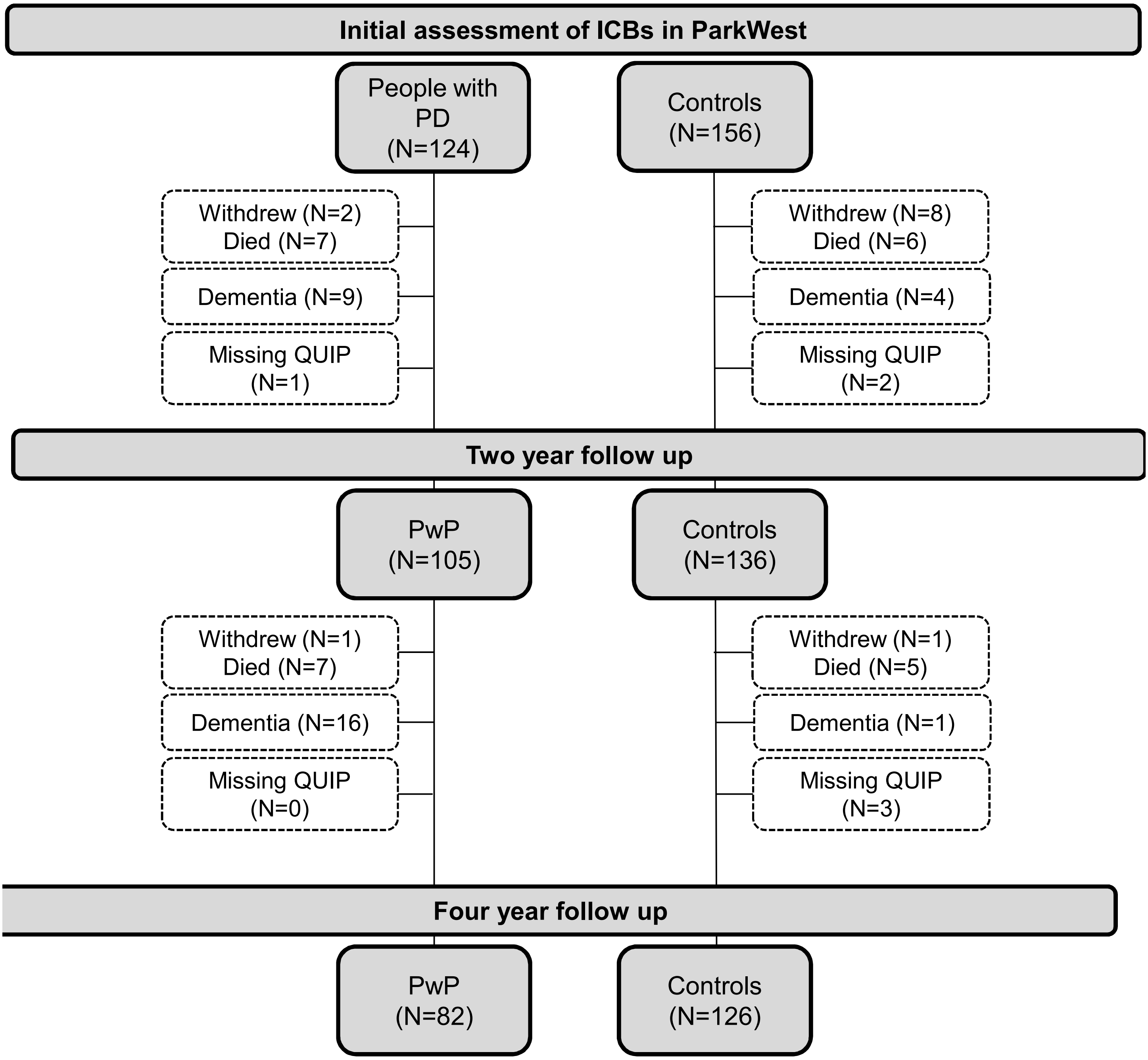
Initial assessment of ICBs in ParkWest
People with PD (N=124)
Controls (N=156)
Withdrew (N=2) Died (N=7)
Dementia (N=9)
Missing QUIP (N=1)
Withdrew (N=8) Died (N=6)
Dementia (N=4)
Missing QUIP (N=2)
Two year follow up
PwP (N=105)
Controls (N=136)
Withdrew (N=1) Died (N=7)
Dementia (N=16)
Missing QUIP (N=0)
Withdrew (N=1) Died (N=5)
Dementia (N=1)
Missing QUIP (N=3)
Four year follow up
PwP (N=82)
Controls (N=126)

Online Resource 2. Frequencies of individual ICBs in PwP over four year follow up

ICB type	Baseline (N=124)	2-year follow up (N=105)	4-year follow up (N=82)
Gambling	2 (1.6)	4 (3.8)	2 (2.4)
Hypersexuality	7 (5.6)	6 (5.7	4 (4.9)
Compulsive shopping	14 (11.3)	11 (10.5)	2 (2.4)
Compulsive eating	6 (4.8)	8 (7.6)	4 (4.9)
Hobbyism	13 (10.5)	15 (14.3)	8 (9.8)
Punding	12 (9.7)	11 (10.5)	6 (7.3)
Walkabout	5 (4.0)	3 (2.9)	1 (1.2)
DDS	3 (2.4)	6 (5.7)	2 (2.4)

Abbreviations: ICBs=Impulsive and compulsive behaviors; PwP=Patients with Parkinson's disease.

All number are N (%).

Online Resource 3. Evolution of ICB symptoms in PD patients over 2 and 4 years of follow-up

	**4-year follow up (N=82)**				**2-year follow up (N=23)**		
**Characteristics**	**No. (%)**	**Baseline**	**2 years**	**4 years**	**No (%)**	**Baseline**	**2 years**
Never ICBs	39 (47.6)	0	0	0	13 (56.5)	0	0
Non-persistent ICBs	7 (8.5)	x	x	0	2 (8.7)	x	0
	3 (3.7)	x	0	x			
	11 (13.4)	x	0	0			
	7 (8.5)	0	x	0			
Persistent ICBs	4 (4.9)	0	x	x	4 (17.4)	x	x
	6 (7.3)	x	x	x			
Uncategorized ICBs	5 (6.1)	0	0	x	4 (17.4)	0	x

Online Resource 4. Results from mixed linear regression models for 4 cognitive domains.

In mixed linear regression models, change in MMSE scores was associated with male gender (B=-1.13, 95 % CI -1.81 – -0.43; P=0.002), higher age (B=-0.09, 95 % CI -0.13 – -0.06; P<0.001) and DA use (1.51, 95 % CI 0.9 – 2.1, P<0.001), but not ICB status. Change in POMP scores for the executive domain was associated with male gender (B=-9.71, 95 % CI -12.72 – -6.70; P<0.001), higher age (B=-0.85, 95 % CI -1.02 – -0.66; P<0.001), follow-up time (B=-2.04, 95 % CI -3.65 – -0.43, P=0.013) and DA use (B=6.21, 95 % CI 3.2 – 9.3, P<0.001), but not ICB status. Change in POMP scores for the attention domain was associated with male gender (B=-12.5, 95 % CI -15.48 – -9.61; P<0.001), higher age (B=-0.65, 95 % CI -0.82 – -0.48; P<0.001) and DA use (5.94, 95 % CI 3.0 – 8.9, P<0.001), but not ICB status. Change in POMP scores for the visuospatial domain was associated with high age (B=-0.54, 95 % CI -0.75 – -0.34; P<0.001), but not ICB status. Lastly, change in POMP scores for the verbal memory domain was associated with male gender (B=-9.71, 95 % CI -12.72 – -6.70; P<0.001), high age (B=-1.10, 95 % CI -1.35 – -0.84; P<0.001), follow-up time (B=-2.56, 95 % CI -5.09 – -0.04, P=0.046), PD duration (B=2.18, 95 % CI 0.87 – 3.49, P=0.001) and DA use (B=8.40, 95 % CI 3.8 – 13.0, P<0.001), but not ICB status.
